# The complete chloroplast genome sequence of *Clerodendrum cyrtophyllum* from Guangzhou, China

**DOI:** 10.1080/23802359.2022.2087549

**Published:** 2022-06-24

**Authors:** Shiqiang Xu, Weizhong Huang, Shike Cai, Jihua Wang

**Affiliations:** a Guangdong Provincial Key Laboratory of Crops Genetics and Improvement, Crops Research Institute, Guangdong Academy of Agricultural Sciences, Guangzhou, PR China; b Guangdong Luofushan Sinopharm Co., Ltd, Huizhou, PR China

**Keywords:** Lamiaceae, *Clerodendrum cyrtophyllum*, chloroplast genome, medicinal plant, phylogenetic analysis

## Abstract

*Clerodendrum cyrtophyllum* is a well-known medicinal plant in southern China. Here, we presented the complete chloroplast (cp) genome of *C. cyrtophyllum* using Illumina high-throughput sequencing technology. The *C. cyrtophyllum* cp genome size is 152,004 bp with 38.13% GC content, including a pair of inverted repeat regions (IR, 51,592 bp) separated by a large single copy (LSC, 86,480 bp) and a small single copy region (SSC, 18,425 bp). It possesses 87 protein-coding genes, 37 *tRNA* genes and eight *rRNA* genes. Phylogenetic analysis fully shows that *C. cyrtophyllum* is closely related to *Clerodendrum bungei* and *Clerodendrum lindleyi*. Overall, the complete cp genome sequence of *C. cyrtophyllum* provides a valuable resource for genetic diversity, phylogenetic relationship, and species identification.

*Clerodendrum cyrtophyllum* Turcz. 1863, belonging to the genus *Clerodendrum* of Lamiaceae family, is a perennial herb that widely distributed in tropical and subtropical regions (Shrivastava and Patel 2007). In Vietnam and China, it has been reported as folk medicine to treat various disease, such as epidemic colds, fever, sore throat, and rheumatic arthritis (Nguyen et al. 2020). Its dried aerial part, called *daqingye* in traditional Chinese medicine, has the effects of clearing heat and detoxification, dispelling wind and eliminating dampness, antioxidant, and anti-inflammatory. It has various phytochemical compounds, such as phenolic acids, polyketides, diterpenes, triterpenes, glycosides, sterols, and flavonoids (Zhou et al. 2013). Among them, phenolic acids, and flavonoids are the main medicinal active ingredients with potent anti-inflammatory and antioxidant effects (Li et al. 2020). However, the same genus species in *Clerodendrum* exhibit high similarity in morphological characters, leading to challenges in species identification. Although studies of *C. cyrtophyllum* using DNA barcodes have been published (Deng et al. 2014), the cp genome has not been assembled. In order to address the cp genome characteristics and evolutionary relationship of *C. cyrtophyllum*, we assembled its complete cp genome by next-generation sequencing technology.

Fresh young leaves of *C. cyrtophyllum* were collected from Guangdong Academy of Agricultural Sciences (Guangzhou, China; N23.1459, E113.3498), and immediately frozen by liquid nitrogen. Genomic DNA was extracted by using plant genomic DNA kit (Omega, Norcross, GA), and deposited in −80 °C refrigerator at Guangdong Provincial Key Laboratory of Crops Genetics and Improvement with the voucher number Dq2020. The herbarium voucher specimen was deposited at the Medicinal Plant Germplasm Resource Nursery (Jihua Wang, wangjihua@gdaas.cn). The paired-end sequencing libraries (insert size 150 bp) were constructed using the DNA Library Fast Construction Kit (Illumina) and sequenced on the Illumina Novaseq platform (Illumina, San Diego, CA) to yield 6,123,816 raw reads. More than 5.0 Gb (base-coverage 525.74×) clean data were obtained by removing adaptors and low-quality reads pairs (*Q* ≤ 20) using Trimmomatic version 0.33 (Golm, Germany) with default parameters (Bolger et al. 2014). GetOrganelle version 1.6.2e (default parameters, Kunming, China) was employed to assemble the *C. cyrtophyllum* cp genome, and Geseq was performed to annotate the complete cp genome with default settings (Tillich et al. 2017; Jin et al. 2020). The final cp genome was evaluated and corrected with the *Clerodendrum japonicum* cp genome as a reference (NCBI accession number: MW222242.1).

The *C. cyrtophyllum* cp genome size was 152,004 bp with 38.13% GC content (GenBank assession number MW858153.1). The structure of the *C. cyrtophyllum* cp genome displayed a typical quadripartite structure with a pair of IRs regions (25,633 bp), which were separated by a LSC (83,413 bp) and a SSC region (17,325 bp). The cp genome of *C. cyrtophyllum* possessed 132 genes, including 87 protein-coding genes, 37 *tRNA* genes, and eight *rRNA* genes. In addition, 17 genes were duplicated in the IR regions and 23 genes contained introns.

To resolve the phylogenetic position of *C. cyrtophyllum*, a phylogenetic analysis is constructed by using the whole cp genome sequences of 16 species in the Lamiaceae family and two outgroups (*Stachys japonica* and *Lamium takeshimense*). The maximum likelihood (ML) phylogenetic tree was inferred using RaxML software version 8.2.12 (Heidelberg, Germany) under the model automatically selected for 1000 bootstraps (Stamatakis 2014). As shown in Figure 1, the bootstrap values of the nodes in the ML tree were 94∼100, and species in the same genus were clustered together, demonstrating a highly robust evolutionary relationship. *C. cyrtophyllum* was clearly clustered with other *Clerodendrum* genus species, and formed a subclade with *C. bungei* and *C. lindleyi*. In conclusion, the characterized cp genome sequence of *C. cyrtophyllum* provides a useful genetic information for the evolutionary relationship and species identification.

**Figure 1.**
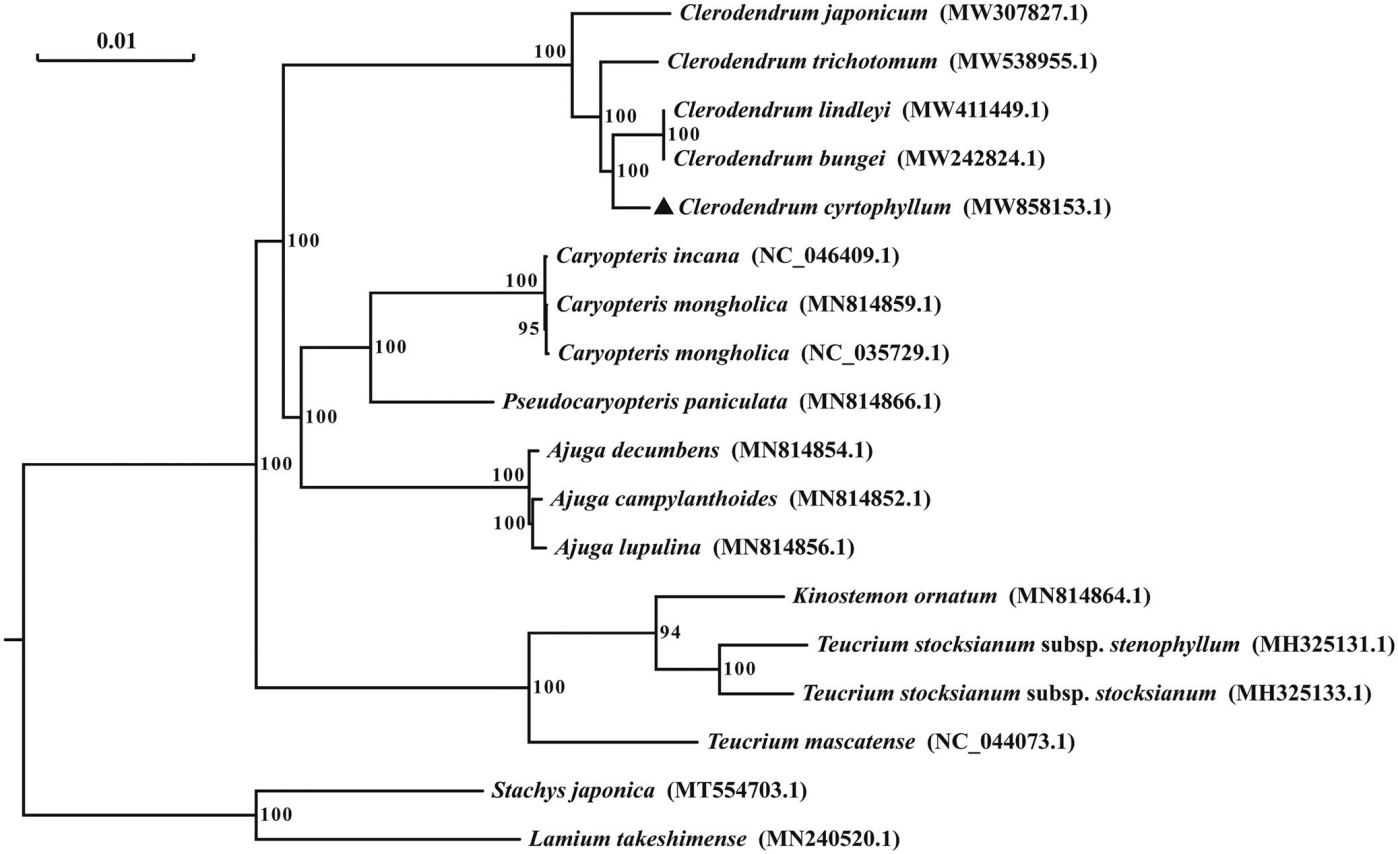
Maximum likelihood phylogenetic tree of *C. cyrtophyllum* with other species based on the complete cp genome sequences. Numbers above the node are bootstrap values based on 1000 replicates.

## Ethics approval

Not applicable.

## Authors’ contributions

Shiqiang Xu: conceptualization, software, writing – original draft. Weizhong Huang: investigation, resources, validation. Shike Cai: project administration, supervision. Jihua Wang: data curation, project administration, writing – review and editing. All authors agree to be accountable for all aspects of the work.

## Data Availability

The genome sequence data that support the findings of this study are openly available in GenBank of NCBI at [https://www.ncbi.nlm.nih.gov/nuccore/MW858153] under the accession no. MW858153. The associated BioProject, SRA, and Bio-Sample number are PRJNA721121, SRR14209297, and SAMN18700176, respectively.
